# Effect of Heterologous Expression of Key Enzymes Involved in Astaxanthin and Lipid Synthesis on Lipid and Carotenoid Production in *Aurantiochytrium* sp.

**DOI:** 10.3390/md23040164

**Published:** 2025-04-11

**Authors:** Yaping Shao, Zhengquan Gao, Fengjie Sun, Yulin Cui, Xinyu Zou, Jinju Ma, Qiaolei Wang, Hao Zhang, Yuyong Wu, Chunxiao Meng

**Affiliations:** 1 School of Pharmacy, Binzhou Medical University, Yantai 264003, China; 15689046125@163.com (Y.S.); gaozhengquan@bzmc.edu.cn (Z.G.); cyl-lc@163.com (Y.C.); 15698067037@163.com (X.Z.); xycrl21@163.com (J.M.); 15689046250@163.com (Q.W.); hao073711@bzmc.edu.cn (H.Z.); 2 Department of Biological Sciences, School of Science and Technology, Georgia Gwinnett College, Lawrenceville, GA 30043, USA; fsun@ggc.edu

**Keywords:** astaxanthin, *Aurantiochytrium* sp., heterologous expression, transcriptome, astaxanthin ester, carotenoid synthesis, lipid synthesis, methanol, ethanol

## Abstract

*Aurantiochytrium* sp., a heterotrophic microorganism, has received increasing attention for its high production of polyunsaturated fatty acids and has been widely applied in various industries. This study intended to optimize the carotenoid synthesis pathway in *Aurantiochytrium* sp. by metabolic engineering to increase the carotenoid content. Multi-sourced key enzyme genes involved in lipid synthesis (*LPAAT* and *DGAT*) and astaxanthin synthesis (*crtZ* and *crtW*) were selected to construct single-gene expression vectors and transformed into *Aurantiochytrium* sp. The results showed that the overexpression of LPAAT of *Phaeodactylum tricornutum* in *Aurantiochytrium* sp. caused an increase of 39.3% in astaxanthin, 424.7% in β-carotene, 901.8% in canthaxanthin, and 575.9% in lutein, as well as a down-regulation of 15.3% in the fatty acid content. Transcriptomics analysis revealed enhanced expression of genes involved in purine and amino acid metabolism in the transformed strains, and the down-regulation of the citric acid cycle led to an increase in the source of acetyl coenzyme A for the production of fatty acids. This study provides strong experimental evidence to support the application of increasing carotenoid levels in *Aurantiochytrium* sp.

## 1. Introduction

*Aurantiochytrium* sp. is a heterotrophic microorganism that is capable of producing lipids up to more than 50% of the dry cell weight. These include docosahexaenoic acid (DHA), a type of omega-3 polyunsaturated fatty acids (PUFAs), which has received increasing attention as an alternative to marine fish oils and accounts for more than 35% of total fatty acids [1,2]. Furthermore, *Aurantiochytrium* sp. is characterized by its synthesis of eicosapentaenoic acid (EPA), rapid growth, and easy commercial production and has become an ideal oil-producing microorganism for the production of both DHA and EPA [3]. Currently, the Food and Drug Administration (FDA) of USA has certified the safety of *Aurantiochytrium* sp. and the resulting DHA oils.

In addition to oils, *Aurantiochytrium* sp. can also synthesize a group of carotenoids, such as β-carotene, canthaxanthin, and astaxanthin [4,5]. As naturally occurring pigments, carotenoids belong to the tetraterpene class of pigments and are widely found in plants and a variety of microorganisms [6,7]. They usually appear yellow, orange, and red in color, and are a natural source of color in many plants and fruits. Their unique molecular structures give carotenoids remarkable antioxidant properties, making them a potent class of antioxidants. Among these carotenoids, astaxanthin has the title of “king of antioxidants”. It has 100 times the antioxidant activity of vitamin E and 10 times the antioxidant activity of lutein and zeaxanthin. Astaxanthin is believed to exert its antioxidant activity by directly quenching and scavenging reactive chemicals, such as reactive oxygen species (ROS) and reactive nitrogen species (RNS) [8]. Natural astaxanthin synthesized by *Aurantiochytrium* sp. has a predominantly all-trans levulinic structure, and is biologically more active than synthetic astaxanthin. This higher bioactivity is attributed to its stereochemical configuration, which enhances its ability to integrate into cell membranes and exert protective effects against oxidative stress [9,10]. Astaxanthin has important biological properties, such as strong antioxidant, anti-inflammatory, and anticancer activities. Its antioxidant capacity is particularly notable, as it can neutralize free radicals and protect cells from oxidative damage, which is implicated in aging, cardiovascular diseases, and neurodegenerative disorders [11,12]. In addition, astaxanthin has been shown to modulate immune responses by enhancing antibody production and reducing inflammation through the inhibition of pro-inflammatory cytokines, such as TNF-α and IL-6 [13,14]. Its anti-cancer potential has also been explored, with studies demonstrating its ability to induce apoptosis in cancer cells and inhibit tumor growth [15,16]. In addition to astaxanthin, canthaxanthin also possesses these same properties. Canthaxanthin, a ketocarotenoid, shares similar antioxidant and anti-inflammatory activities, making it valuable in both nutraceutical and cosmetic applications [17]. These properties give them significant potential in a number of areas, such as antioxidant, anti-inflammatory, immune-enhancing, skin-protective, and bioavailability properties. For instance, astaxanthin has been widely used in skincare products due to its ability to protect the skin from UV-induced damage and improve skin elasticity and moisture retention [18,19]. Moreover, its bioavailability is enhanced when esterified with fatty acids, as the esterified form improves stability and absorption in the human body [20]. Furthermore, due to its strong antioxidant properties and intense orange-red coloration, it is used in aquaculture as a feed and colorant for Atlantic salmon and trout, giving the species the characteristic pink color. In aquaculture, astaxanthin is essential for the growth, survival, and pigmentation of farmed fish, and its inclusion in feed has been shown to improve fish health and product quality [21]. Beyond aquaculture, astaxanthin is also utilized in the food industry as a natural colorant and preservative, as well as in the pharmaceutical industry for its therapeutic potential [22].

Currently, commercially available carotenoids are mainly derived from chemical synthesis [23], which typically utilizes petroleum-based feedstocks, chemical reagents, and catalysts as raw materials to mimic the naturally occurring process [24]. Although it is highly efficient and cost effective in production and accounting more than 80% of the carotenoid market, several disadvantages of chemical synthesis of carotenoids, such as by-product safety issues and low oxidizability, pose potential risks to human health [25]. In addition to chemical synthesis, another common source of carotenoid production is plants, such as carrots and pumpkins [26,27,28]. They are often considered “natural” and is attractive to consumers who seek natural foods. However, plant extraction methods have the disadvantages of high cost, seasonal limitation, cumbersome extraction process, etc., making them difficult to maintain stable production throughout the year. With the rapid advances in biotechnology and metabolic engineering tools, microbial fermentation is gaining favor as a more environmentally friendly and sustainable production method for carotenoid production [29,30]. For example, a group of microorganisms have been commonly used to synthesize carotenoids [31,32,33], showing main advantages of independence on factors such as land and climate and the potential to reduce environmental pollution [34]. With the continuous advancement of technology and the gradual reduction in production costs, microbial fermentation is expected to be widely used in the future, especially in the fields of food, nutraceuticals, and natural cosmetics.

As an important industrial microorganism, *Aurantiochytrium* sp. has played an important role in the production of DHA in recent years, gradually attracting the attention of the scientific and industrial communities in the field of carotenoid production. The cells of *Aurantiochytrium* sp. use glucose as a carbon source to produce acetyl coenzyme A via the glycolytic pathway [35]. Subsequently, acetyl coenzyme A enters the mevalonate (MVA) pathway, a key metabolic pathway for isoprenoid biosynthesis, to generate geranylgeranyl diphosphate (GGPP), a key precursor of the carotenoid synthesis pathway. GGPP is converted to β-carotene through a series of enzymatic reactions, and a hydroxyl group (–OH) is added to the carbon atoms of β-carotene catalyzed by the key enzyme hydroxylase (CRTZ). Then, β-carotene ketolase (CRTW) catalyzes the conversion of hydroxyl groups on the carotenoid molecule to ketone groups (C=O), ultimately generating astaxanthin, the end product of the carotenoid metabolic pathway [36]. During their synthesis of carotenoids, such as lutein, canthaxanthin, and astaxanthin, they all form carotenoid esters under the appropriate conditions. For example, astaxanthin-DHA monoesters and other esterified forms are stored in lipid droplets in cells of *Aurantiochytrium* sp. [37]. This is mainly due to the capability of *Aurantiochytrium* sp. to synthesize a variety of unsaturated fatty acids, especially the omega-3 fatty acids (e.g., DHA), which are normally found in triglycerides (TAGs) and phospholipids [38]. The production of carotenoid esters not only increases the stability of carotenoids but also improves their bioavailability. In addition to the above pathways involved in carotenoid synthesis, the rate-limiting enzymes involved in the lipid synthesis pathway, i.e., lysophosphatidic acid acyltransferase (LPAAT) and acylglycerol acyltransferase (DGAT), may also indirectly regulate the pigment levels. It has been reported that TAGs may interact and coordinate with astaxanthin biosynthesis through astaxanthin esters [39]. During TAG-fatty acid synthesis, LPAAT acts as a rate-limiting enzyme catalyzing the conversion of lysophosphatidic acid (LPA) to phosphatidic acid (PA), which is not only involved in the generation of key intermediates of signal transduction and cellular lipid biosynthesis, but also critical for TAG synthesis [40,41]. Similarly, DGAT is the rate-limiting enzyme in the synthesis of TAGs, catalyzing the acylation of diacylglycerol (DAG) [42]. Previous studies have demonstrated that the addition of diacylglycerol acyltransferase (DGAT) to an in vitro enzyme activity assay system significantly inhibits the net synthesis of both astaxanthin and its esterified forms. This inhibitory effect is likely attributed to the role of DGAT in diverting fatty acyl-CoA substrates toward triglyceride synthesis, thereby reducing the availability of fatty acids for esterification with astaxanthin. This observation aligns with the established metabolic competition between lipid biosynthesis and carotenoid esterification, where the enzymatic activity of DGAT preferentially channels metabolic flux toward neutral lipid production at the expense of carotenoid esterification [43]. Therefore, DGAT is considered a candidate enzyme for the biosynthesis of astaxanthin esters [44].

The aims of this study were to optimize the synthetic pathways of carotenoids and lipids in *Aurantiochytrium* sp. and to explore the underlying synthetic mechanisms by means of metabolic engineering. Currently, the industrial production of natural carotenoids faces critical challenges, such as high cost and low yield. However, microorganisms, such as *Aurantiochytrium* sp., have shown significant potential for lipid and carotenoid production. Enhancing the efficiency of their carotenoid synthesis in these microorganisms has become an important research direction. Our study provided new perspectives to investigate the mechanisms of carotenoid synthesis and to advance the potential of *Aurantiochytrium* sp. for applications in metabolic engineering and industrial production, especially in natural product synthesis.

## 2. Results

### 2.1. Expression Vector Construction and Molecular Characterization of Exogenous Genes Expressed in Aurantiochytrium sp.

**Figure 1:**
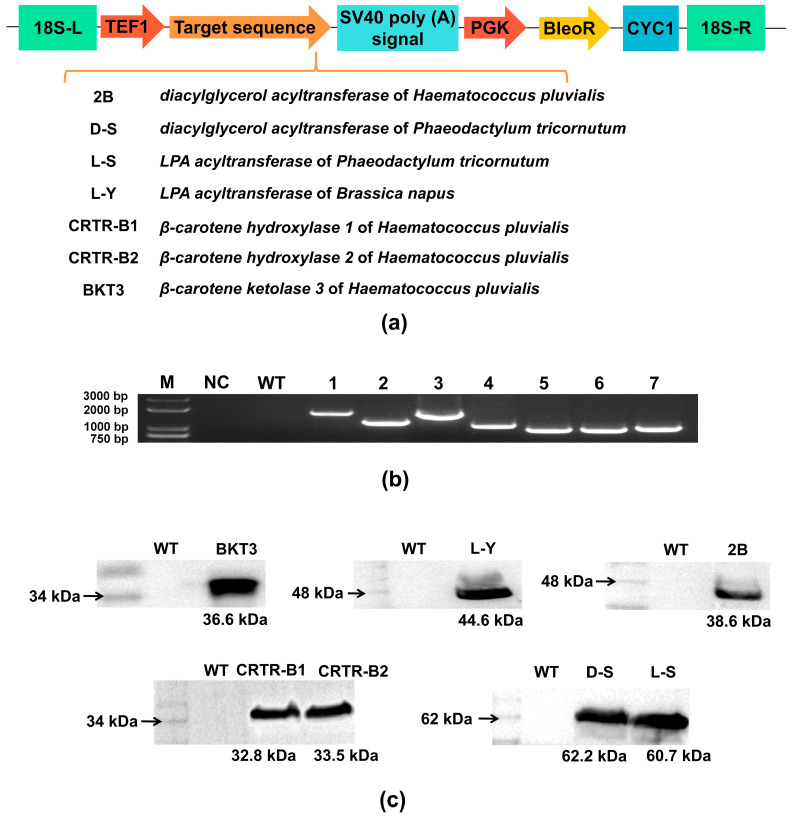
Transformation validation of *Aurantiochytrium* sp. (**a**) Schematic representation of expression vector for exogenous genes expressed in 7 strains of *Aurantiochytrium* sp. (**b**) PCR analysis of exogenous genes. M: DL8000 maker; NC: negative control; WT: wild type; lanes 1–7 represent positive monocolonies of strains L-S, L-Y, D-S, 2B, CRTR-B1, CRTR-B2, and BKT3, respectively. (**c**) Western blotting analysis of strains L-S, L-Y, D-S, 2B, CRTR-B1, CRTR-B2, and BKT3.

The ORFs of the amino acid coding regions of the genes of *Haematococcus pluvialis* (*DGAT*, *BKT3*, *CRTR-B1*, and *CRTR-B2*), *Phaeodactylum tricornutum* (*DGAT* and *LPAAT*), and *Brassica napus* (*LPAAT*) were homologously recombined into the pWG-18s-Bleor vector, respectively. The exogenous genes were regulated by promoter TEF1. The screening marker gene *BleoR* was promoted by PGK promoter and CYC1 terminator (Figure 1a).

The genomic DNA of the monocolonies of *Aurantiochytrium* sp. strains was further characterized. All exogenous genes were analyzed by PCR to identify the positive monocolonies. The results showed that among the positive monocolonies, the *LPA acyltransferase* gene (strain L-S) and *diacylglycerol acyltransferase* gene (strain D-S) of *Phaeodactylum tricornutum* were detected as about 1629 bp and 1194 bp bands, respectively; the *LPA acyltransferase* gene (strain L-Y) of *Brassica napus* was about 1584 bp; and the *diacylglycerol acyltransferase* gene (strain 2B), *β-carotene hydroxylase 1* (strain CRTR-B1) and 2 (strain CRTR-B2) genes, and *β-carotene ketolase 3* gene (strain BKT3) of *Haematococcus pluvialis* were about 1045 bp, 861 bp, 861 bp, and 978 bp, respectively (Figure 1b). The sequence lengths of the amplified fragments were consistent with the integration of the exogenous genes. Western blotting analysis showed that protein fragments of L-S (60.7 kDa), L-Y (44.6 kDa), D-S (62.2 kDa), 2B (38.6 kDa), CRTR-B1 (32.8 kDa), CRTR-B2 (33.5 kDa), and BKT3 (36.6 kDa) were detected in positive monocolonies, respectively, whereas the corresponding bands were not detected in WT (Figure 1c). These results indicated that the exogenous genes were successfully expressed in the transformant strains of *Aurantiochytrium* sp.

To evaluate the efficiency of the electroporation technique for transforming *Aurantiochytrium* sp., plasmid DNA at a concentration of 800 μg μL^−1^ was used. On average, 15 colonies were obtained per transformation attempt and 1 or 2 of these colonies were successfully transformed, i.e., a transformation efficiency of approximately 1–2 transformants per 800 μg of plasmid DNA. These results supported electroporation as a feasible method for genetic transformation of *Aurantiochytrium* sp. strains, while the efficiency may require further optimization for high yields.

### 2.2. Physiological and Biochemical Characterization of Positive Monocolonal Strains of Aurantiochytrium sp.

**Figure 2:**
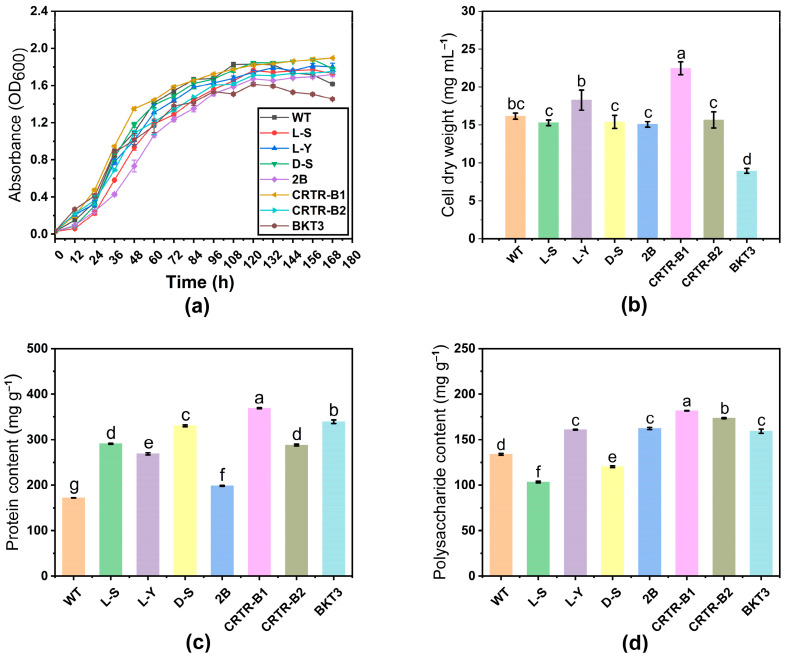
Physiological and biochemical analyses of wild type (WT) and transformant strains of *Aurantiochytrium* sp. (**a**) Absorbance. (**b**) Dry cell weight. (**c**) Protein content. (**d**) Polysaccharide content. Data are expressed as mean ± standard deviation (SD) of 3 biological replicates. Different lowercase letters “a, b, c, d, e, f, and g” represent significant differences between treatments (*p* < 0.05).

To assess the effect of exogenous genes on host cells, culture (100 mL) of each *Aurantiochytrium* sp. strain was inoculated in the fermentation medium, and the growth status of each strain was evaluated by the measurement of OD_600_ every 12 h from the initial inoculation to sugar depletion. The growth curves of the positive transformants and WT of *Aurantiochytrium* sp. in 7 d (i.e., reaching glucose depletion in the shake flask) showed that when the strains were cultured to the end of sugar depletion, the cell densities of most strains were higher than that of WT, except for strain BKT3 (Figure 2a), with the highest cell density (OD_600_ = 1.897) detected in strain CRTR-B2, while the cell density of WT reached OD_600_ of 1.616. The highest growth rate of *Aurantiochytrium* sp. was observed at 84 h, when the cell densities of the positive transformant strains were lower than that of WT.

Overall, the growth patterns were largely consistent among all transformant strains and WT *Aurantiochytrium* sp., i.e., 0–12 h was the growth retardation period and 12–84 h was the logarithmic growth period. During the fermentation period (84–132 h), most strains entered the stabilization phase and the strains BKT3 and L-Y entered the decline phase at 132–168 h. The strains reached the stable phase or even the decline phase during sugar depletion and started to enter nutrient stress (Figure 2a). These results indicated the effects of the introduction of exogenous genes on host growth. The growth of the strains was inhibited in the early stages of growth. However, with the gradual depletion of sugar, the cell densities of most positive monocolonal cells became higher than that of WT, except for the strain BKT3. The biomass of the organisms on the last day of fermentation was comparatively evaluated using dry cell weight method (Figure 2b). Strains L-Y and CRTR-B1 accumulated more biomass (i.e., dry cell weight), reaching 1.1 and 1.4 times higher than that of WT, respectively, whereas the dry cell weight of strain BKT3 was significantly lower than that of WT.

Proteins play multiple roles in the physiological functions, infection mechanisms, and adaptation to environmental changes of *Aurantiochytrium* sp., and are important components for maintaining cell survival and reproduction. All the transformants of *Aurantiochytrium* sp. showed a significant increase in protein content, compared to WT. The highest increase in protein content was detected in strain CRTR-B1, which was accounted for 36.9% of the dry cell weight and was 2.1 times higher than that of WT. Strains BKT3 and D-S showed a 2.0-fold and 1.9-fold increase in the protein content, respectively (Figure 2c). The variations in the contents of intracellular biomolecules of these transformants were further characterized. As important cellular components of *Aurantiochytrium* sp., polysaccharides have shown multiple functions in energy storage, structural support, signal transduction, and immunity, reflecting the physiological state of the cells [45]. Our results showed that the polysaccharide contents of transformants were significantly higher than that of WT (133.8 mg g^−1^), except for L-S and D-S (Figure 2d). The greatest increase in polysaccharide content was observed in strain CRTR-B1, accounting for 18.2% of the dry cell weight, which was 1.4 times higher than that of WT. This was followed by strain CRTR-B2, accounting for 17.3% of the dry cell weight, which was 1.3 times higher than that of WT. However, strains L-S and D-S showed a decrease of 22.7% and 10.2% in polysaccharide content, compared to WT, respectively. These results indicated that exogenous genes significantly affected the polysaccharide content of different transformants of *Aurantiochytrium* sp.

### 2.3. Effect of Exogenous Genes on Carotenoid Content of Aurantiochytrium sp.

**Figure 3:**
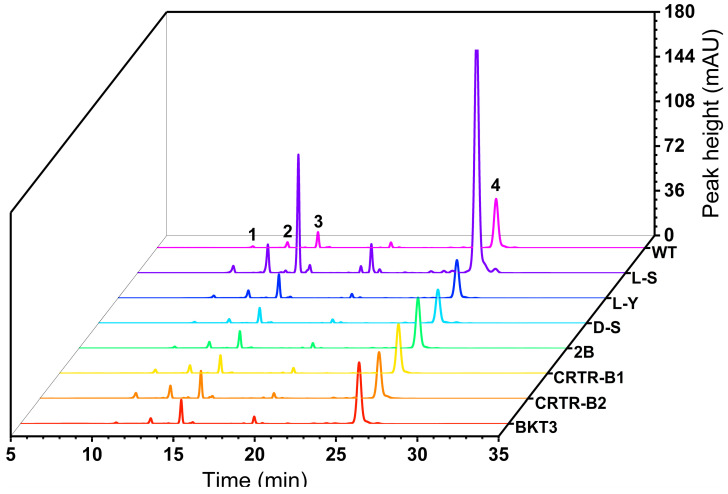
High-performance liquid chromatographic detection of pigments in shake flask fermentation of wild type (WT) and seven transformed strains of *Aurantiochytrium* sp. Peak 1, astaxanthin; peak 2, lutein; peak 3, canthaxanthin; peak 4, β-carotene.

**Figure 4:**
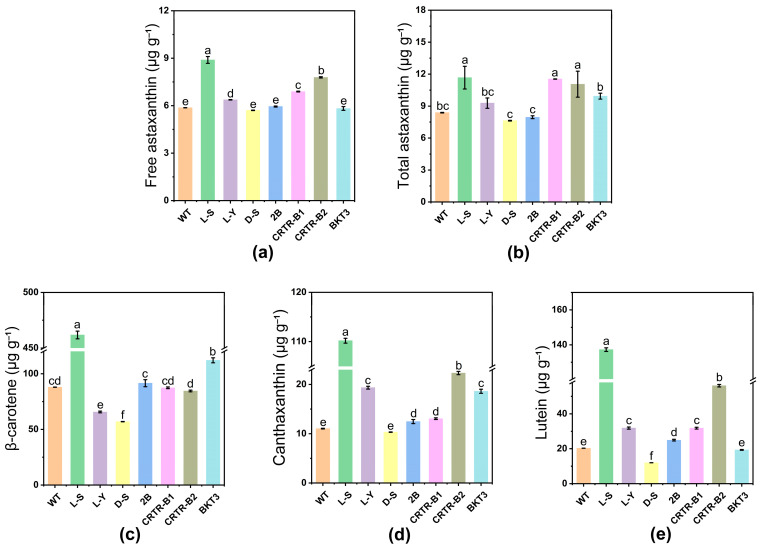
High performance liquid chromatographic detection of carotenoid content of wild type (WT) and transformant strains of *Aurantiochytrium* sp. (**a**) Free astaxanthin. (**b**) Total astaxanthin. (**c**) β-carotene. (**d**) Canthaxanthin. (**e**) Lutein. Data are expressed as mean ± standard deviation (SD) of 3 biological replicates. Different lowercase letters “a, b, c, d, e, and f” represent significant differences between treatments (*p* < 0.05).

The introduction of exogenous genes caused no alteration in the carotenoid composition of *Aurantiochytrium* sp., i.e., astaxanthin, β-carotene, and canthaxanthin were the main carotenoids detected in *Aurantiochytrium* sp. (Figure 3). Among these, β-carotene was involved in the production of canthaxanthin in the presence of both hydroxylase and β-carotene ketolase, and canthaxanthin was ultimately used to produce free astaxanthin. Strains L-S, L-Y, CRTR-B1, and CRTR-B2 showed significant differences in the content of free astaxanthin, compared to WT. The free astaxanthin contents of strains L-S and CRTR-B2 reached 8.9 ± 0.2 μg g^−1^ and 7.8 ± 0.0 μg g^−1^, which were enhanced by 50.8% and 32.2%, respectively (Table 1; Figure 4a). The free astaxanthin molecules are esterified with fatty acids through the reaction between the hydroxyl group (–OH) of astaxanthin and the carboxyl group (–COOH) of fatty acids. These astaxanthin esters are then stored in cytoplasmic lipid droplets. Through enzymatic digestion and extraction, astaxanthin esters can be converted to free astaxanthin, ultimately obtaining both free astaxanthin and the total astaxanthin. Our results showed that the total astaxanthin contents of strains L-S, CRTR-B1, and CRTR-B2, were significantly higher than that of WT. The highest increase was detected in strain L-S, reaching 11.7 ± 1.1 μg g^−^^1^, which was an increase of 39.3%, compared to WT. This was followed by strains CRTR-B1 and CRTR-B2, showing an increase of 36.9% and 32.1%, respectively, compared to WT (Table 1; Figure 4b). Strains CRTR-B1 and BKT3 showed the largest increase in the content of astaxanthin esters by 88.0% and 64.0%, respectively, compared to WT (Table 1).

The results of the contents of β-carotene revealed significant difference in the contents of β-carotene in strains 2B, BKT3, and L-S, compared to WT (Table 2). The highest increase in the β-carotene content was detected in strain L-S, reaching 461.7 μg g^−^^1^, which was an increase of 424.7%, compared to WT (88.0 μg g^−1^). Strain BKT3 showed an increase of 27.3%, whereas both strains L-Y and D-S showed significant decrease in β-carotene content (Table 2; Figure 4c).

Canthaxanthin is a key carotenoid produced in this study, and its biosynthesis pathway has been extensively optimized to achieve improved yields. The results demonstrate that our engineered strain significantly enhances canthaxanthin production compared to natural microorganisms. The contents of canthaxanthin in most transformant strains of *Aurantiochytrium* sp., except for strain D-S, showed significant changes, compared to WT (11.0 μg g^−1^). The canthaxanthin contents of strains L-S and CRTR-B2 reached 110.2 μg g^−1^ and 22.3 μg g^−1^, respectively, which were 901.8% and 102.7% higher than that of WT (Table 2; Figure 4d). Strains L-Y, 2B, CRTR-B1, and BKT3 also showed a significant increase in canthaxanthin content, compared to WT. In addition to the changes in pigments mentioned above, variations in the content of lutein, another key member of the carotenoid metabolic pathway, are also noted (Figure 4e). The results showed that, except for strain BKT3, the lutein content in the transformant strains exhibited significant changes compared to WT. The L-S strain showed the highest lutein content at 137.2 μg g^−1^, which was 6.8 times that of WT. The strain CRTR-B2 showed a lutein content of 56.1 μg g^−1^, which was 2.8 times that of WT. In contrast, the D-S strain showed a significant decrease in lutein content. These results further demonstrate the varied effects of genetic modifications on carotenoid biosynthesis.

### 2.4. Detection of Fatty Acid Composition in Strain L-S of Aurantiochytrium sp.

**Figure 5:**
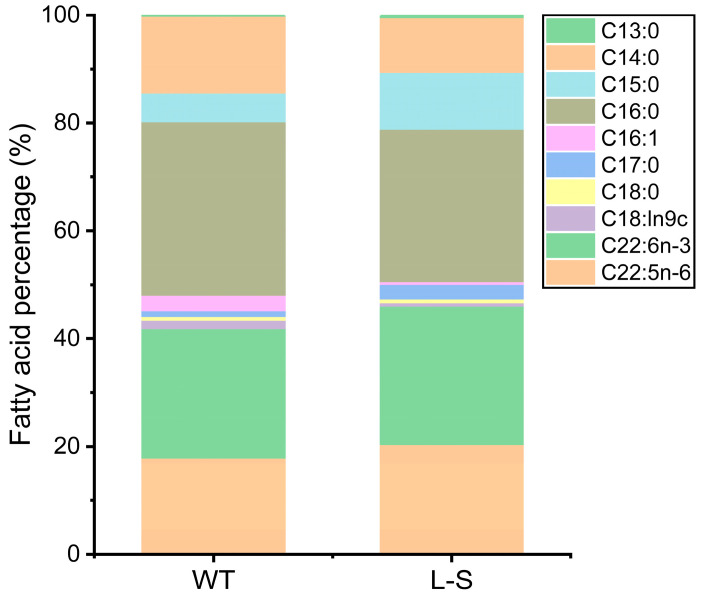
Contents of fatty acids in wild type (WT) and strain L-S of *Aurantiochytrium* sp. All values are expressed as mean ± standard deviation (SD) (n = 3 biological replicates).

LPAAT is considered a rate-limiting enzyme that acetylates LPA to generate PA, which is further involved in triacylglycerol synthesis. To further investigate the effect of LPAAT on the fatty acid levels in *Aurantiochytrium* sp., strains L-S and WT were chosen to further explore the variations in the fatty acid content by fat extraction and fatty acid methylation. The results showed that a total of six saturated fatty acids (C13:0, C14:0, C15:0, C16:0, C17:0, and C18:0) and four unsaturated fatty acids (C16:1, C18:ln9c, C22:5n-6 and C22:6n-3) were detected in both strain L-S and WT (Figure 5). The highest level in the content of saturated fatty acids was detected in C16:0, followed by C14:0 and C15:0, while C22:6n-3 (DHA) showed the highest level in the content of unsaturated fatty acids, followed by C22:5n-6 (DPA n-6). Among the saturated fatty acids, the C16:0, C14:0, and C15:0 contents of strain L-S reached 9.9 g/100 g, 3.5 g/100 g, and 3.7 g/100 g, respectively. Among the unsaturated fatty acids, the contents of DHA and DPA n-6 in strain L-S were 9.0 g/100 g and 7.1 g/100 g, respectively. In addition, the ratio of DHA/DPA n-6 in strain L-S was approximately the same as that observed in WT. Overall, it was evident that C16:0, DPA n-6 and C22:6n-3 were the three main fatty acids of *Aurantiochytrium* sp. Strain L-S revealed a reduction by 15.3% in the total amount of fatty acids, especially in C14:0 and C16:0, which were decreased by 40.7% and 25.6%, respectively, compared to WT. However, the fatty acid content of strain L-S was enriched in saturated fatty acids, such as C13:0, C15:0, and C17:0, with C17:0 reaching 1.0 g/100 g, which was 2.0 times higher than that of WT. This was followed by C13:0, which was 1.9 times higher than that of WT. The fatty acid composition (%) in strain L-S and WT revealed that in strain L-S, the proportion of C16:0 was decreased from 32.2% to 28.3%, which was a decrease of 12.1%, whereas the proportion of C22:6n-3 was increased from 24.0% to 25.7%, an increase of 7.1%, compared to WT. Moreover, the DPA n-6 proportion of strain L-S was increased by 12.8%, compared to WT. A significant change was also detected in the proportion of other two types of abundant saturated fatty acids, C14:0 and C15:0. The content of C14:0 was decreased from 14.3% to 10.0%, a decrease of 30.1%, while the content of C15:0 was increased from 5.3% to 10.6%, an increase of 100.0%. These results showed that with the introduction of LPAAT from *Phaeodactylum tricornutum* into *Aurantiochytrium* sp., the fatty acid ratio in the transformant strains of *Aurantiochytrium* sp. was altered and the relative abundance of unsaturated fatty acids was increased.

### 2.5. Transcriptome Analysis of Wild Type and Strain L-S of Aurantiochytrium sp.

**Figure 6:**
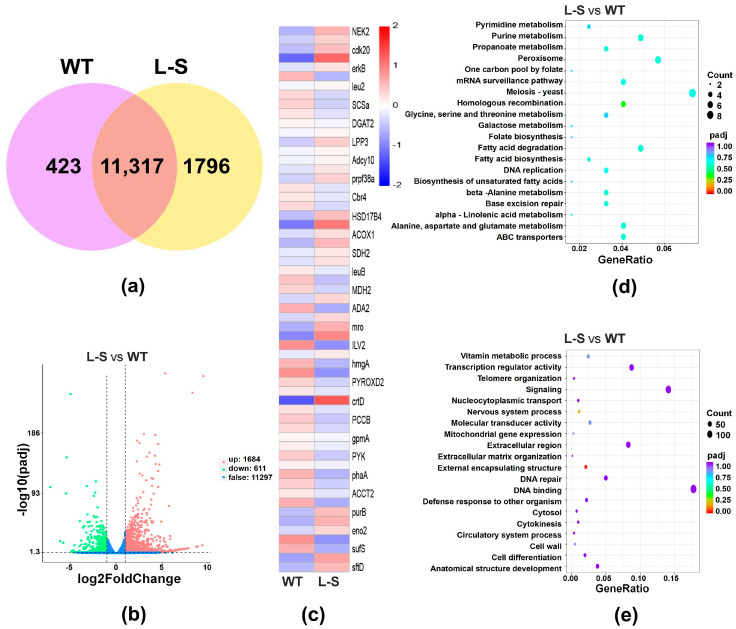
Differential expression analysis of genes between strain L-S (the experimental group) and wild type (WT; the control group) of *Aurantiochytrium* sp. under shake flask fermentation to sugar depletion. (**a**) Venn diagram showing the differentially expressed genes (DEGs) based on strain L-S and WT. (**b**) Volcano plot of DEGs. (**c**) Heatmap of expression of 30 genes involved in carotenoid-related metabolic pathways. (**d**) KEGG enrichment analysis based on up-regulated DEGs. (**e**) GO enrichment analysis based on up-regulated DEGs.

To further investigate the effects of the introduction of *LPAAT* gene from *Phaeodactylum tricornutum* to *Aurantiochytrium* sp., transcriptomic analysis was performed based on strain L-S (the experimental group, which contained the highest carotenoid content) and WT (the control group) of *Aurantiochytrium* sp., each of three biological replicates. The raw sequences obtained were subjected to raw data filtering, sequencing error rate checking, GC content distribution checking, and quality evaluation. Q20 values were higher than 98% and Q30 values were higher than 95.5% in both strain L-S and WT groups. The Pearson’s correlation coefficient (R2) was greater than 0.93, indicating a high degree of similarity in the expression patterns between the samples and reliable sequencing data. A total of 11,317 co-expressed genes were detected in both strain L-S and WT cultured to the end of sugar depletion (Figure 6a), with a total of 2295 genes showing significant differences in expression levels (1684 up-regulated and 611 down-regulated) (Figure 6b). To further explore the biological functions of these differentially expressed genes (DEGs), these DEGs were mapped onto carotenoid-related metabolic pathways, showing that the DEGs were mainly enriched for purine metabolism, MAPK signaling pathway, amino acid metabolism, tricarboxylic acid (TCA) cycle, and fatty acid biosynthesis (Figure 6c). Both GO and KEGG enrichment analyses were performed based on up-regulated DEGs to further identify the functions of these DEGs. The results of KEGG analysis showed the significantly enriched metabolic pathways related to fatty acid synthesis included purine metabolism (enriched by 6 DEGs), fatty acid biosynthesis (enriched by 4 DEGs), and unsaturated fatty acid biosynthesis (enriched by 2 DEGs) (Figure 6d), as well as several amino acid metabolic pathways (enriched by 10 DEGs). The results of GO analysis showed that the top 20 significantly enriched GO terms were mainly associated with the categories of cellular component (CC) and biological process (BP) (Figure 6e). These results indicated that the up-regulated DEGs in strain L-S were predominantly involved in pathways related to fatty acid metabolism and cellular function, compared to WT, highlighting the potential role of these pathways in the biological characterization of strain L-S of *Aurantiochytrium* sp. The transcriptome data were uploaded to the National Center for Biotechnology Information (NCBI; https://www.ncbi.nlm.nih.gov/; accessed on 19 January 2025) database (accession number: PRJNA1212494).

**Figure 7:**
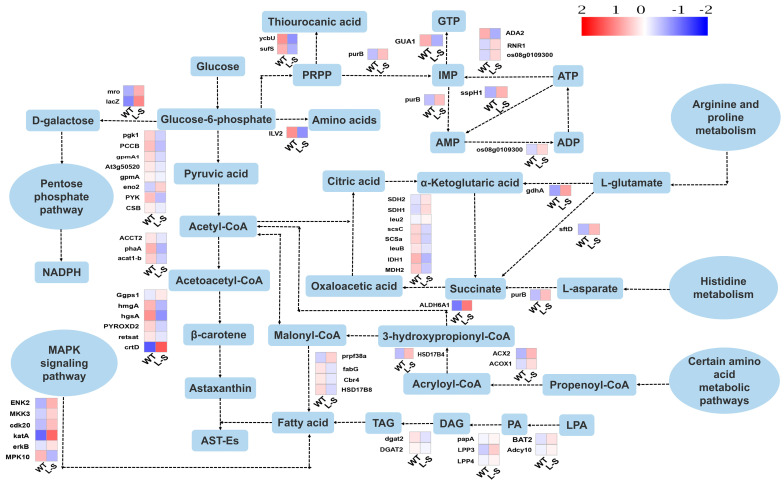
Transcriptomic mapping of various metabolic pathways involved in carotenoid and fatty acid synthesis based on strain L-S and wild type (WT) of *Aurantiochytrium* sp. AMP: adenosine 5′-monophosphate; IMP: inosine monophosphate; ADP: adenosine 5′-diphosphate; ATP: adenosine 5′-triphosphate; PRPP: phosphoribosyl diphosphate; GTP: guanosine 5′-triphosphate; LPA: lysophosphatidic acid; PA: phosphatidic acid; DAG: diacylglycerol; TAG: triacylglycerol; AST-Es: astaxanthin esters; purB: adenylosuccinate lyase; ADA2: adenosine deaminase; RNR1: ribonucleoside-diphosphate reductase alpha chain; Os08g0109300: adenylate kinase; ENK2: serine/threonine-protein kinase CLA4; MKK3: mitogen-activated protein kinase; cdk20: cyclin-dependent kinase; katA: catalase; erkB and MPK10: p21-activated kinase 1; PYK: pyruvate kinase; CSB and eno2: enolase; gpmA, At3g50520, and gpmA1: phosphoglycerate mutase; PCCB: phosphoglycerate kinase; pgk1: 3-phosphoglycerate kinase; prpf38a: fatty acid desaturase; fabG, Cbr4, HSD17B8, and HSD17B4: β-Ketoacyl-ACP reductase; dgat2 and DGAT2: diacylglycerolacyltransferase; papA, LPP3, and LPP4: phosphatidic acid phosphatase; BAT2 and Adcy10: lysophosphatidate acyltransferase; sspH1: nuclease; GUA1: GMP synthase; ycbU: aminotransferase; sufS: proteolysis; mro: mutarotase; lacZ: β-galactosidase; ILV2: thiamine pyrophosphate; gdhA: glutaminase; ACOX1 and ACX2: Acyl-CoA oxidase; ALDH6A1: dehydrogenase; Ggps1: geranylgeranyl pyrophosphate synthase; ACCT2, phaA, and acat1-b: acetyl-CoA acetyltransferase; hmgA: 3-hydroxy-3-methyl glutaryl coenzyme A reductase; hgsA: hydroxymethylglutaryl-CoA synthase; PYROXD2, retsat, and crtD: phytoene desaturase; SDH1 and SDH2: succinate Dehydrogenase; leu2: aconitase; scsC and SCSa: succinyl-CoA Synthetase; leuB and IDH1: isocitrate dehydrogenase; MDH2: malate dehydrogenase.

The effects of *LPAAT* overexpression on fatty acid biosynthesis in *Aurantiochytrium* sp. was further investigated (Figure 7). It was observed that some of the amino acid metabolic pathway products were directly involved in the fatty acid synthesis pathway, and the gene expression of *acyl coenzyme A oxidase* and *malonate semialdehyde dehydrogenase* were up-regulated 2.2-fold and 4.4-fold, respectively, directly or indirectly regulating the levels of two fatty acid synthesis precursors, acetyl coenzyme A and malonyl coenzyme A, respectively. Purine metabolism generates ATP to provide energy for fatty acid synthesis. The transcriptomics analysis showed that *adenylate kinase* (*AK*), a key enzyme for ATP generation, was significantly up-regulated 1.9-fold, and the expression of *adenylosuccinate lyase* (*ASL*) was significantly higher in strain L-S than that of the control group. In addition, gene expression of *GMP synthase* was significantly down-regulated 2.4-fold, which implied that IMP attenuated the flow of GMP metabolism pathway. Moreover, the IMP precursor phosphoribosyl diphosphate (PRPP) decreased the expression of *ycbU* and *sufS* by 3.4-fold and 2.9-fold, respectively, compared with WT, during metabolism of thiourocanic acid, suggesting that PRPP attenuated the alternative metabolic pathways. Transcriptomic results of strain L-S showed that the expression of dehydrogenases (encoded by *succinate semialdehyde dehydrogenase* and *glutamate dehydrogenase*) or cleaving enzymes (encoded by *adenylosuccinate lyase*) responsible for the synthesis of alpha-ketoglutarate or succinic acid based on glutamate and aspartate was significantly up-regulated 2.2, 2.8, and 2.1-fold, respectively, compared with WT. Moreover, transcriptomic analysis of lipid biosynthesis showed that the gene expression of *LPAAT* and *phosphatase* were up-regulated and *diacylglycerol acyltransferase* (*DGAT*) expression was down-regulated in strain L-S, compared to WT. In addition, in the MAPK signaling pathway, *serine/threonine protein kinase* (*CLA4*), *cyclin-dependent protein kinases* (*CDKs*), and *mitogen-activated protein kinase* (*MEK*) were up-regulated 2.4-fold, 2.1-fold, and 12.4-fold, respectively, and *peroxidase* (*PRX*) was up-regulated 5.3-fold, compared to WT, indicating increased activity of the MAPK signaling pathway (Figure 7).

The biosynthesis of astaxanthin as a secondary metabolite of *Aurantiochytrium* sp. was also investigated. Interestingly, the expression of most genes encoding enzymes involved in glycolysis and TCA cycle was down-regulated. For example, *phosphofructokinase* (*pfk*) was down-regulated 1.7-fold and *phosphoglycerate kinase* (*PGK*) was down-regulated 1.8-fold in glycolysis pathway. The expression of *geranylgeranyl diphosphate synthase* (*GGPS*) was up-regulated 1.3-fold, catalyzing the generation of geranylgeranyl pyrophosphate (GGPP) from mevalonate, which was the starting metabolite of the carotenoid synthesis pathway [46]. Phytoene synthase (PSY), a key enzyme in the carotenoid metabolism pathway, was expressed at an elevated level and up-regulated 6.4-fold. The transcriptomic analysis revealed a significant decrease in the expression levels of three genes, i.e., *phosphoglycerate mutase* (*At3g50520*) involved in glycolysis, *phosphoribosyl-AMP cyclohydrolase* (*his7*) related to amino acid metabolism, and *S-adenosylmethionine synthetase* (*METK3*) playing a key role in the synthesis of S-adenosylmethionine. These findings were further confirmed by qPCR (Figure S1), showing the consistent expression patterns of *At3g50520*, *his7*, and *METK3* and suggesting that thee down-regulation of these genes was closely associated with fatty acid biosynthesis.

## 3. Discussion

To explore the application potential of *Aurantiochytrium* sp., we optimized the production of carotenoids and lipids by introducing exogenous genes to generate transformant strains of *Aurantiochytrium* sp. with enhanced accumulation of carotenoids and lipids. In recent years, increasing the carotenoid content in cells of *Aurantiochytrium* sp. has attracted significant attention. We focused on two pathways regulating the production of carotenoids. First, the direct regulatory pathway, i.e., the carotenoid synthesis pathway, with both hydroxylase and ketolase as the rate-limiting enzymes for astaxanthin biosynthesis. Second, the indirect pathway, i.e., the TAG-fatty acid cycle, which plays an important role in regulating the quantity and quality of carotenoid esters, ultimately promoting the biosynthesis and accumulation of carotenoids.

### 3.1. Effect of Exogenous Genes on Protein and Polysaccharide Accumulation in Aurantiochytrium sp.

Our study revealed variations in protein and polysaccharide levels in transgenic strains of *Aurantiochytrium* sp. with overexpression of genes *DGAT*, *LPAAT*, *crtZ*, or *crtW*. The introduction of exogenous genes increased the protein contents in host cells (Figure 2c). These results were in agreement with previous studies, showing increased protein level in transgenic soybean seeds [47]. The enhanced protein content was likely due to the overexpression of *DGAT* and *LPAAT*, which affected the lipid storage and cellular energy status of *Aurantiochytrium* sp. and indirectly promoted the synthesis and secretion of proteins. However, hydroxylase and ketolase overexpressing strains of *Aurantiochytrium* sp. could alter the protein levels through the involvement of their intermediate products in cellular signaling pathways due to the direct involvement of these two enzymes in carotenoid metabolic pathway. Among the mutant strains, CRTR-B1 exhibited the most significant increase in protein content. Although CRTR-B1 and CRTR-B2 are isoforms, their protein content differs significantly, which may be attributed to overall metabolic changes in the cells. Specifically, the heterologous expression of CRTR-B1 could redirect metabolic flux towards cellular components, resulting in higher protein content. In contrast, the metabolic flux in CRTR-B2 may have been directed more towards carotenoid biosynthesis, explaining its lower protein content but potentially higher carotenoid production. These findings suggest that the differences in protein content between CRTR-B1 and CRTR-B2 are likely due to metabolic flux redistribution rather than the higher expression of specific enzymes. Further studies are necessary to verify these speculations. In addition, our results revealed that the polysaccharide content of strains L-S and D-S was significantly lower than that of WT (Figure 2d). This was probably due to the efficient expression of triglyceride synthesis-related genes (*DGAT* and *LPAAT*) of *Phaeodactylum tricornutum* in strains L-S and D-S of *Aurantiochytrium* sp., ultimately directing the metabolic pathway of the cells toward lipid synthesis, leading to a decrease in the levels of precursors and energy used for polysaccharide synthesis, and reducing polysaccharide synthesis.

### 3.2. Effect of Exogenous Genes on Biomass and Carotenoid Accumulation in Aurantiochytrium sp.

Our study revealed that the overexpression of exogenous genes caused significant changes in growth and pigmentation content of *Aurantiochytrium* sp. Compared with WT, the cell growth of strains L-Y and CRTR-B1 was increased 1.1-fold and 1.4-fold, respectively (Figure 2b). These results were consistent with previous studies, showing that *crtZ* transgenic tobacco plants gained increased biomass [48]. In addition, the dry cell weight of *Chlamydomonas reinhardtii* with overexpression of exogenous gene *β-carotene hydroxylase* was significantly increased [49]. Furthermore, it has been reported that LPAAT overexpression showed no significant difference in the growth of transformants of *Neochloris oleoabundans* during exponential growth, whereas the overall growth was lower than that of WT [50]. This observation was consistent with the findings of strains L-S and L-Y revealed in our study. Interestingly, the growth of the BKT3 mutant strain was significantly reduced compared to WT, which could be attributed to increased metabolic activity due to the heterologous expression of the *bkt3* gene. This could lead to excessive consumption of cellular resources (e.g., energy and precursors), ultimately inhibiting cell growth. This observation is consistent with previous studies. For example, Li et al. [51] found that heterologous expression of *AtPAP2* suppressed plant growth in tomatoes, and Zhang et al. [52] reported that heterologous expression of *SrERF5* inhibited root growth in *Arabidopsis*. Additionally, the *bkt3* gene might exert a toxic effect on *Aurantiochytrium* sp., further suppressing its growth. This hypothesis is supported by the findings of Raimundo et al. [53], demonstrating that the expression of *ppIAPP* exerted toxic effects on yeast growth and cell viability.

Furthermore, our study revealed significant changes in the levels of carotenoids in the transformant strains of *Aurantiochytrium* sp. The carotenoid content in strain L-S was significantly changed, compared to WT. Specifically, the contents of astaxanthin, β-carotene, and canthaxanthin were increased by 39.3%, 424.7%, and 901.8%, respectively (Figure 4). In addition, the astaxanthin and canthaxanthin contents in the hydroxylase-overexpressing strains (CRTR-B1 and CRTR-B2) and the ketolase-overexpressing strain (BKT3), both key enzymes in the carotenoid metabolism pathway, showed significant changes compared to WT, suggesting their critical roles in modulating carotenoid biosynthesis. For example, the total astaxanthin content of strain CRTR-B1 and CRTR-B2 was increased by 36.9% and 32.1%, respectively, compared with WT. Strain BKT3 also showed a significant increase in the content of β-carotene and canthaxanthin. These results were consistent with previous studies, showing that overexpression of β-*carotene hydroxylase* gene significantly increased astaxanthin production in *Aurantiochytrium limacinum*, and the content of canthaxanthin was enhanced in transformant strains of *A. limacinum* with overexpression of ketolase [54]. In addition, co-expression of both *CrtZ* and *CrtW* increased the astaxanthin production in *Escherichia coli* [55].

In addition to genetic modifications, environmental factors, such as light, temperature, and nutrient availability are well known to significantly affect the production of metabolites. For example, Maltsev et al. [56] found that light intensity can significantly regulate the production of fatty acids and carotenoids. Furthermore, Diaz-MacAdoo et al. [57] demonstrated that blue light significantly stimulates the synthesis of lutein and other carotenoids. Moreover, Sekova et al. [58] showed that an increase in temperature leads to significant changes in the content of unsaturated fatty acids in *Yarrowia lipolytica*. We also investigated the effects of methanol and ethanol as inducers on carotenoid production. The results are shown in Figure S2, demonstrating that changes in nutrient conditions can significantly alter the composition of metabolites. These results indicate that the addition of methanol increased astaxanthin accumulation, while ethanol increased canthaxanthin production. In addition, nitrogen-deficient environments can promote lipid accumulation in algae and inhibit cell growth [59]. This provides new insights into the improvement of metabolite yields by optimizing environmental conditions.

### 3.3. Effect of LPAAT on Fatty Acid Composition in Aurantiochytrium sp.

To assess the effect of *LPAAT* overexpression on the fatty acid profiles of *Aurantiochytrium* sp., variations in fatty acid composition were detected by GC. Our results suggested that heterologous expression of *LPAAT* altered the fatty acid profile in *Aurantiochytrium* sp. (Figure 5), resulting in an increase in the proportion of C22:6n-3, DPA n-6 and C15:0 and a decrease in the proportions of C16:0 and C14:0 in fatty acids. These results are consistent with previous studies, showing that LPAAT plays a critical role in regulating fatty acid composition and lipid metabolism [60,61]. Additionally, studies on *Aurantiochytrium* sp. have demonstrated that genetic modifications targeting lipid biosynthesis pathways can significantly alter fatty acid profiles, including the accumulation of odd-chain fatty acids, such as C15:0 [62,63]. Moreover, these results were consistent with the previous study, showing significant effect of *LPAAT* overexpression on the fatty acid composition in the transgenic strains of *Phaeodactylum tricornutum*, and on FA synthesis and accumulation of long-chain FAs with chain lengths of C14–C20, i.e., causing an increase in the degree of unsaturation in the exponential phase [64]. Furthermore, overexpression of *LPAAT* could lead to altered fatty acid distribution in lipids of kale-type oilseed rape seeds [65]. However, previous studies showed that although overexpression of *LPAAT* affected the fatty acid content in *Chlamydomonas reinhardtii*, cotton, and tobacco, variations were detected in the fatty acid changes among different taxa [66,67]. Wayne et al. found that soybean LPAAT expression resulted in the redistribution of DHA-containing TAG species and that transgenic expression of *Schizochytrium* sp. or soybean LPAAT increased the proportion of DHA at the sn-2 position of the TAG and the total amount of DHA accumulated in the seed oil of oilseed plants [68]. Starikov et al. reported that *Synechococcus elongatus* cells expressing exogenous LPAAT synthesized 26% of C14:0 [69]. Furthermore, in *Arabidopsis*, overexpression of PrLPAAT4 resulted in a significant increase in oleic acid content in seeds [70]. These studies suggested that LPAAT functioned in a species-dependent manner [71,72]. In addition, it has also been shown that overexpression of LPAAT paralogous homologs has different preferences for fatty acid accumulation in the same species. For example, Ogawa et al. analyzed two LPAAT paralogs, PlsC4 and PlsC5, from the choleretic bacterium *Shewanella livingstonensis* Ac10 and found that PlsC4 preferred isotridecanoic acid, whereas PlsC5 preferred palmitoleic acid [73].

The expression of exogenous LPAAT may influence the intracellular regulatory network by altering the levels of PA or other lipid intermediates, thereby modulating the expression of fatty acid synthesis-related genes. This regulatory shift could potentially lead to a change in the substrate preference of the fatty acid synthase (FAS) system, favoring the incorporation of propionyl-CoA (a precursor for odd carbon chain fatty acids) over acetyl-CoA [74,75]. As a result, the synthesis of odd carbon chain fatty acids (e.g., 15:0 and 17:0), which are typically produced at low levels, was significantly increased in the L-S strain. Additionally, LPAAT may enhance the activity of specific acyltransferases, facilitating the incorporation of odd carbon chain fatty acid precursors (e.g., 15:0 and 17:0) into PA or downstream lipid synthesis pathways. This effect could be attributed to LPAAT’s ability to alter its substrate specificity, favoring odd carbon chain acyl-CoAs, or to its role in regulating the activity of other enzymes involved in the fatty acid synthesis pathway, such as propionyl-CoA carboxylase or fatty acid elongases [76,77]. These mechanisms collectively promote the accumulation of odd carbon chain fatty acids in the L-S strain. Our results are consistent with previous studies, e.g., Li et al. found that over-expression of *LPAAT* gene in *Phaeodactylum tricornutum* increased fatty acid chain length [64], while Woodfield et al. reported that LPAAT could alter the distribution of fatty acids in seed lipids [65].

Notably, the DHA/DPA n-6 ratio remained largely unchanged in strain L-S, compared to WT, indicating that the relative balance between these two key unsaturated fatty acids was maintained despite the overall changes in fatty acid composition. Although the absolute content of DPA n-6 was slightly decreased in strain L-S, its proportion in the total fatty acid composition was increased, suggesting that overexpression of LPAAT may selectively affect the synthesis or accumulation of specific unsaturated fatty acids. It is possible that the metabolic fluxes of DHA and DPA n-6 synthesis may be coordinately regulated through shared enzymes or precursors in the PUFA biosynthetic pathway.

In our study, strain L-S showed significant changes not only in fatty acid composition but also in the total amount of fatty acids. Combined with the changes in carotenoid production, it is hypothesized that the flow of carbon sources was redirected, with more carbon directed toward the carotenoid synthesis pathway. It is speculated that this is likely due to the fact that the fatty acid pathway shared key metabolic precursors (e.g., acetyl coenzyme A or malonic acid) with the carotenoid synthesis pathway, thereby facilitating pigment accumulation [78]. Changes in the content and percentage of certain saturated and unsaturated fatty acids and suggest that there may be a balance between PUFA synthesis and the production of other lipids. This balance might be further influenced by the competition for acetyl-CoA, a common precursor for both fatty acid and carotenoid biosynthesis. The observed increase in carotenoid content could thus be a direct consequence of the metabolic shift induced by LPAAT overexpression, i.e., more carbon molecules are allocated to the carotenoid pathway at the expense of partial fatty acid synthesis. This observation aligns with previous findings in *Thraustochytrids*, where metabolic engineering strategies have been shown to simultaneously enhance both lipid and carotenoid production by modulating shared precursor pools [79]. Additionally, the changes in DHA and DPA n-6 levels could indirectly affect carotenoid production by modulating membrane fluidity and oxidative stress responses [80].

### 3.4. Differentially Expressed Genes Involved in Carotenoid Synthesis in Aurantiochytrium sp.

To assess the potential effect of *LPAAT* overexpression on carotenoid production in *Aurantiochytrium* sp., comparative transcriptomics analysis was performed based on strain L-S and WT of *Aurantiochytrium* sp. In the main carotenoid synthesis pathway, *Aurantiochytrium* sp. underwent glycolysis, MVA, and carotenoid metabolism to generate the non-lipid secondary metabolites, i.e., astaxanthin, β-carotene, and canthaxanthin [81]. Interestingly, the transcriptomic analysis revealed that most of the genes involved in the glycolytic pathway were down-regulated, suggesting that as glucose was gradually consumed in the incubation process, glycolysis was gradually weakened [82]. These results were consistent with previous studies, showing that the cells favored the synthesis of fatty acids and citric acid, whereas fatty acids inhibited glycolysis by modulating the activities of phosphofructokinase and pyruvate kinase, and citric acid acted on glycolysis by directly inhibiting phosphofructokinase [83,84]. In addition, up-regulation of PSY and GGPS, both the controlling enzymes for carotenoid production in carotenoid synthesis pathway, could contribute to the precursor synthesis levels of carotenoids and the final yield of carotenoids [85,86].

### 3.5. Differentially Expressed Genes Involved in the Production of Unsaturated Fatty Acids and ATP Synthesis in Aurantiochytrium sp.

Carotenoids in *Aurantiochytrium* sp. exist in different forms, e.g., free state or esterified state [37]. The esterified state is formed when carotenoids combine with unsaturated fatty acids to form esters. Unsaturated fatty acid accumulation was dependent on a continuous supply of ATP and acetyl coenzyme A [87,88]. ATP, as the main energy source in cellular processes, not only provides energy to support the synthesis of acetyl coenzyme A, but also plays a crucial role in the activation of the fatty acid synthase complex during the extended reaction of fatty acid synthesis [89,90]. Moreover, the availability of ATP is tightly regulated by cellular energy status, and its synthesis is closely linked to the metabolic demands of the cell, particularly during periods of high lipid biosynthesis. Purine metabolism was one of the main pathways for ATP production. Our study showed that the expression of PRPP in the ATP anabolic process was elevated, while its expression in other metabolic processes was decreased in the experimental group (i.e., strain L-S). This was probably due to the increased cellular demand for fatty acids, and cells prioritized the flow of energy toward ATP, and to maintain the balance of total purine nucleotide levels in the cell, they regulated the expression of genes involved in GTP synthesis. The elevated ATP supply could not only support the enhanced acetyl coenzyme A production but also directly affect the regulation of key enzymes, such as acetyl coenzyme A carboxylase (ACC), which catalyzes the first step in fatty acid synthesis. These shifts in metabolic flux could contribute to the increased fatty acid esterification with carotenoids, thereby affecting carotenoid accumulation. Furthermore, feedback mechanisms play a pivotal role in modulating metabolic flux. For instance, the accumulation of esterified carotenoids and fatty acids indicates a demand for more precursors, thereby up-regulating the expression of genes involved in acetyl-CoA and malonyl-CoA production [91]. This feedback loop ensures a dynamic balance between precursor availability and end-product accumulation, optimizing the metabolic flux toward fatty acid synthesis and carotenoid esterification.

### 3.6. Differentially Expressed Genes Involved in the Production of Unsaturated Fatty Acids and Acetyl Coenzyme A Synthesis in Aurantiochytrium sp.

TCA cycle plays an important role when acetyl coenzyme A is supplied for fatty acid synthesis. Amino acids are key backfill metabolites in the TCA cycle, undergoing deamination or dehydrogenation processes to produce α-ketoglutarate or succinate, and ultimately citric acid [92,93]. Intermediates of the TCA cycle, such as citric acid, could act as metabolic regulators and cofactors for fatty acid synthesis [94]. Studies have shown that citric acid was transported from the mitochondria to the cytoplasm via endosomal transport and was subsequently hydrolyzed by citrate lyase to acetyl coenzyme A and oxaloacetic acid [89]. The acetyl coenzyme A was used as a precursor for fatty acid synthesis, while oxaloacetic acid was eventually converted to pyruvate [95]. The shift in the TCA cycle flux, particularly the reduced activity of certain enzymes, likely increased the levels of citric acid and acetyl coenzyme A, thereby enhancing fatty acid synthesis. In our study, the expression of genes encoding enzymes involved in the formation of citric acid was up-regulated, whereas the expression of genes encoding most enzymes in citric acid cycle was down-regulated. This suggests a metabolic shift toward lipid biosynthesis, where the cells redirect resources from energy production to the synthesis of storage lipids. It is hypothesized that the weakened center of the TCA cycle and the elevated activity of the citrate transport process toward the endosomes led to enhanced levels of both acetyl coenzyme A and pyruvate. In addition, citric acid stimulated the activity of acetyl coenzyme A carboxylase, which catalyzed fatty acid synthesis. These speculations were consistent with previous reports, showing that down-regulation of the citric acid cycle led to an increase in the level of acetyl coenzyme A for fatty acid production, ultimately enhancing lipid accumulation [96,97]. Furthermore, the observed metabolic changes may reflect an adaptive response to environmental or nutritional cues, optimizing resource allocation for lipid storage. Moreover, previous studies demonstrated that MAPK signaling played an important role in lipid anabolism [98]. This was in accordance with the findings revealed in our study, showing that CLA4, MEK, and CDKs further stimulated the genes expression of *fatty acid synthase* (*FAS*) and *acetyl coenzyme A carboxylase* (*ACC*), enhancing fatty acid synthesis. The integration of MAPK signaling with metabolic pathways ensures coordinated regulation of fatty acid biosynthesis, highlighting the importance of signaling networks in modulating metabolic flux. These findings highlight the association between signaling pathways and metabolic processes in regulating lipid and carotenoid biosynthesis in *Aurantiochytrium* sp.

### 3.7. Potential Limitations and Future Directions of the Work

Compared to other natural microorganisms producing carotenoids and lipids, our engineered strains are largely more productive, while further improvements are necessary to surpass the high productivity of established industrial strains. For instance, the carotenoid and lipid yields of our engineered Aurantiochytrium sp. strains are higher than those of WT Aurantiochytrium sp. and some other natural producers, but lower than those of highly optimized industrial strains (e.g., Haematococcus pluvialis for carotenoids and Schizochytrium sp. for lipids) [99,100]. However, our approach offers a unique advantage by enabling the co-production of both DHA and carotenoids, showing significant potential for industrial applications. Although the current yield has not yet reached commercial production standards, our results demonstrate the significant potential of metabolic engineering for improving carotenoid production in microorganisms. For example, the astaxanthin yield achieved in this study is significantly higher than that of natural microorganisms reported in the literature. Nevertheless, further optimization is still needed to meet the demands of large-scale industrial production. The main limitations of our approach include: (1) metabolic burden caused by heterologous pathway expression, which may affect cell growth and overall productivity [101,102]; (2) potential accumulation of intermediate metabolites that could inhibit the metabolic flux [103]; and (3) the need for further optimization of substrate utilization efficiency to reduce production costs. Scaling up the production process to an industrial level presents several challenges, such as ensuring consistent performance under large-scale fermentation conditions, optimizing oxygen transfer and mixing efficiency, and developing cost-effective downstream processing methods for carotenoid extraction and purification. Additionally, the stability and robustness of the engineered strains under industrial conditions need to be comprehensively evaluated. Compared to other natural microorganisms producing carotenoids, our engineered strains are more productive, while further improvements are necessary to surpass the productivity of established industrial strains. Future studies will focus on optimizing metabolic pathways through systems biology and synthetic biology tools (e.g., CRISPR-Cas9 and dynamic regulation systems) [104], improving culture conditions, such as the carbon-to-nitrogen ratio and the addition of specific precursors (e.g., methanol or ethanol), which have been shown to significantly enhance carotenoid accumulation, and enhancing strain robustness and productivity through adaptive laboratory evolution (ALE) [105,106].

While this study focuses on optimizing carotenoid production, the safety of the final product is equally important. The engineered strains are designed to operate under controlled conditions, minimizing risks associated with contamination or unintended by-products. Future studies will include a comprehensive safety assessment, following guidelines such as those provided by the European Food Safety Authority (EFSA), to ensure the suitability of these strains for consumers and industrial applications.

## 4. Materials and Methods

### 4.1. Culture of Aurantiochytrium sp.

*Aurantiochytrium* sp. strain CCAP 4062/3 was obtained from Tianjin Institute of Industrial Biotechnology (Tianjin, China) and cultured at 28 °C and 150 rpm in fermentation medium [80 g L^−^^1^ glucose, 4 g L^−^^1^ yeast extract, 15 g L^−^^1^ seawater crystal (a salt mixture obtained by evaporating seawater), 2 g L^−^^1^ KCl, 1 g L^−^^1^ CaCl_2_·2H_2_O, 5 g L^−^^1^ MgSO_4_·7H_2_O, and 6.4 g L^−^^1^ MgCl_2_] in a shaking incubator (Zhichu, Shanghai, China).

### 4.2. Plasmid Construction

According to the structure of single gene overexpression vector, the plasmids were constructed by the single fragment homologous recombination method. The target sequence was amplified using the synthetic plasmid (Sangon Biotech, Shanghai, China) as a template based on the specific primers (Table S1; Tsingke Biotech, Beijing, China), and the long vector frame was amplified using the pMG-18S-Bleor plasmid as a template based on specific primers (Table S1; Tsingke Biotech, Beijing, China). The construction of the transformation vector pMG-18s-Bleo-1 was finally completed using ClonExpress Ultra one-step cloning kit (Vazyme, Nanjing, China). Plasmid extraction was performed using GoldHi EndoFree Plasmid Midi Kit (CWBIO, Taizhou, Jiangsu, China).

### 4.3. Transformation of Aurantiochytrium sp.

The plasmids were introduced into cells of *Aurantiochytrium* sp. using electrotransformation. Briefly, cells of *Aurantiochytrium* sp. cultured to logarithmic phase were collected by centrifugation at 5000 rpm and 4 °C for 10 min. Then, pre-cooled distilled water was added first to resuspend and collect the cells, and then pre-cooled 1 M sorbitol was added to resuspend and collect the microorganisms. The above process was repeated twice. Then, an appropriate amount of 1 M sorbitol and 10 μL of plasmid DNA (1000 ng μL^−^^1^) were added and kept in an ice bath for 5 min. Subsequently, the mixture was transferred to a pre-cooled electrowinning cup for 10 min. The multifunctional electroporator was used for electroporation with the electric field strength set at 2.0 KV, the pulse time at 0.2 ms, and the pulse distance at 2 mm. After confirming the successful electroporation, the mixture was added with appropriate amount of activation medium and resuscitated at 150 rpm and 28 °C for 10 h. All experiments were performed in 3 biological replicates. The resuscitated cells were coated and screened on plates with bleomycin (Solarbio^®^, Beijing, China) resistance for 3 d. Single colonies were picked and screened for 3 generations by streaking in resistant solid medium. Purified monocolonies were incubated in liquid medium at 28 °C and 180 rpm.

### 4.4. Molecular Characterization of Transformant Strains of Aurantiochytrium sp.

The exogenous genes of the transformants of *Aurantiochytrium* sp. were characterized using PCR and Western blot analyses to verify the successful recombination and expression of the exogenous fragments in the transformed strains of *Aurantiochytrium* sp. at the gene and protein levels. *Aurantiochytrium* sp. cells were collected by high-speed centrifugation for genomic DNA extraction using SiO_2_ milling combined with DNA extraction buffer (Solarbio^®^, Beijing, China), a standard method widely used for efficient and reliable DNA isolation. The wild type (WT) *Aurantiochytrium* sp. was used as a blank control. PCR was performed using primer pair PMG-F/PMG-R (Table S1), and the amplified fragments were sequenced to verify the gene sequences. Cells from positively transformed strains were collected, resuspended using cell lysate, and treated in phenylmethylsulfonyl fluoride (PMSF). The suspension was treated with sonication and centrifuged at high speed to collect the proteins, which were further concentrated using ultrafiltration tubes and analyzed for protein expression using Western blotting analysis.

### 4.5. Measurement of Physiological and Biochemical Indices of Aurantiochytrium sp.

In our previous study, we observed significant fluctuations in carotenoid content specifically during the sugar depletion phase. This observation suggested that sugar depletion likely play a critical role in regulating carotenoid biosynthesis. Therefore, to better understand the molecular mechanisms underlying the carotenoid biosynthesis, we selected this specific time point to perform the expression analysis.

Positive monocolonies and WT of *Aurantiochytrium* sp. were fermented in fermentation medium in shake flasks at 150 rpm/min and 28 °C until the depletion of sugar, and the optical density (OD_600_) was measured every 12 h. Cells were collected at the end of sugar depletion, and biomass was determined using the dry weight method. Briefly, the collected cells were vacuum freeze-dried to a constant weight and then the biomass was calculated.

*Aurantiochytrium* sp. was assayed for biochemical indicators (i.e., levels of proteins and polysaccharides) to investigate the effect of exogenous genes on their production. The powder (50 mg) was weighed and added with PBS and PMSF for extraction and ultrasonically treated for 10 min using a cell crusher at 30% power. The supernatant was collected by centrifugation to determine the protein content using BCA Protein Quantification Kit (Vazyme, Nanjing, China). The sample (40 mg) was added to 20 times the volume of distilled water and vortexed to mix well. The solution was ultrasonicated for 30 min to disrupt cell walls and release polysaccharides into the aqueous phase, followed by extraction at 70 °C for 3 h to further enhance the solubility of polysaccharides. This step was repeated 3 times to ensure complete extraction, and the supernatant was collected and combined by centrifugation at 8000× *g* for 10 min to remove insoluble debris. The centrifugation parameters were optimized to achieve a clear supernatant while minimizing the loss of polysaccharides. The crude polysaccharide was obtained by concentrating the supernatant using a vacuum rotary evaporator at 60 °C to reduce the volume, followed by precipitation using 95% ethanol (final ethanol concentration of 70%) at 4 °C overnight. Ethanol precipitation is a widely used method for polysaccharide isolation, as it selectively precipitates polysaccharides while leaving smaller molecules (e.g., monosaccharides and oligosaccharides) in solution. The precipitated polysaccharides were then collected by centrifugation at 8000× *g* for 15 min, washed twice with 70% ethanol to remove impurities, and dried under vacuum to obtain the crude polysaccharide extract. The use of 70% ethanol for washing ensured the removal of residual sugars and other contaminants without significant loss of polysaccharides. The carbohydrate content in the crude polysaccharide was determined by the phenol-sulfuric acid method [107], which is a widely accepted and reliable assay for quantifying total carbohydrates. In brief, a known amount of the crude polysaccharide was dissolved in sterile water and mixed well. Then, 5% phenol solution and concentrated sulfuric acid were added sequentially to the sample. The reaction was carried out at room temperature for 30 min to allow complete color development. The absorbance of the resulting solution was measured at 490 nm using a spectrophotometer. To ensure accuracy, each sample was analyzed in triplicate, and the results were averaged to minimize experimental error.

### 4.6. Determination of Carotenoid Content in Aurantiochytrium sp.

The powder (0.2 g) was weighed and dissolved in acetone solution, shaking vigorously for a few sec, and treated with an ultrasonic crusher using an ultrasonic power of 40% for 30 min. The supernatant was collected by high-speed centrifugation at 4 °C and assayed for the levels of four pigments (i.e., free astaxanthin, canthaxanthin, lutein, and β-carotene).

The above supernatant solution (1 mL) was used for total astaxanthin extraction. The solution was added with acetone solution and 0.1 M Tris-HCl buffer (pH = 7.0), mixed thoroughly, and then heated in a water bath at 37 °C for 2 min. Cholesterol esterase solution was then added for the enzymatic reaction at 37 °C for 1 h. At the end of the reaction, petroleum ether and anhydrous sodium sulfate were added and shaken vigorously to perform extraction experiments. Briefly, the solution was placed in a refrigerator at 4 °C for 10 min to stratify the solution, and the upper layer of the solution was collected. This extraction process was repeated several times until the upper solution became colorless. All the collected pigment samples were blown dry using a nitrogen blower, then added with acetone for dissolution, and finally the total astaxanthin content was assayed using high performance liquid chromatograph (HPLC).

### 4.7. Analysis of Fatty Acid Composition of Aurantiochytrium sp.

This study strictly followed the Chinese National Standard GB 5009.168–2016 (available at: https://sppt.cfsa.net.cn:8086/db, accessed on 13 September 2024), Method 2 (external standard method) for fatty acid analysis. The samples underwent hydrochloric acid hydrolysis, followed by extraction with an ether-petroleum ether mixed solution, and subsequent saponification and methylation under alkaline conditions to ultimately produce fatty acid methyl esters (FAMEs). The separation and detection analyses were performed using gas chromatography, and the quantification of individual fatty acid methyl ester content was performed using the external standard method. The FAMEs were analyzed using a Shimadzu gas chromatograph coupled with a Sigma SP2560 capillary column (100 m × 0.25 mm × 0.2 μm). Detection conditions were as follows: flow rate, 1 mL min^−1^; hydrogen flow rate, 30 mL min^−1^; air flow rate, 300 mL min^−1^; split ratio, 5:1; gradient heating conditions. The content was determined by integration using GC software, with calculations based on the national standard GB 5009.168–2016 (i.e., the external standard method of the second method).

### 4.8. Transcriptome Analysis

Transcriptome analysis was performed based on WT and transformant strain of *Aurantiochytrium* sp. with the highest carotenoid content using high-throughput RNA sequencing (Novogene, Tianjin, China). Samples of the transformed and WT strains were collected during the sugar depletion phase and RNA was extracted. Total RNA was extracted from these samples and subjected to stringent quality control using the Agilent 2100 Bioanalyzer (Santa Clara, CA, USA) to ensure RNA integrity (RIN > 7). mRNA was then enriched from the total RNA, and sequencing libraries were constructed following the standard Illumina protocol. High-throughput sequencing was performed on the Illumina platform. The raw sequencing data were quality controlled using FastQC [108] software to remove low-quality reads and splice sequences to obtain clean reads, which were de novo assembled into transcriptomes using Trinity software [109]. Subsequently, the assembled transcripts were aligned to the reference genome of *Saccharomyces cerevisiae* for further annotation and enrichment analysis. Differential gene expression analysis was performed using the DESeq2 software package. The data were normalized to screen for significantly differentially expressed genes (DEGs). Next, Gene Ontology (GO) enrichment analysis was performed based on the up-regulated DEGs using GOseq [110] to calculate the degree of enrichment for each GO term based on the Wallenius non-central hypergeometric distribution model. Significantly enriched GO entries were calculated and compared in the whole genomic context. KEGG pathway enrichment analysis was performed based on up-regulated DEGs using KOBAS software [111]. The levels of KEGG pathway enrichment were assessed by hypergeometric test method. Padj is the *p* value after correction for multiple hypothesis testing. Padj takes the value in the range of 0–1, and the closer the value is to zero, the more significant the enrichment is. The enrichment results were compared with the entire annotated gene set to determine the relevance of each pathway to carotenoid biosynthesis and metabolism. The results of GO and KEGG enrichment analyses were visualized using relevant tools, such as Bubble Plots, to illustrate the enrichment of specific GO terms and KEGG pathways. To verify the accuracy of the transcriptome data, we performed quantitative real-time PCR (qRT-PCR) using primers listed in Table S2. Expression levels of key DEGs were assessed in parallel with transcriptome data to confirm the robustness of the findings.

### 4.9. Statistical Analysis

SPSS 22 software was used to analyze the experimental data, which were expressed as mean ± standard deviation (SD) (n = 3 biological replicates). One-way ANOVA was used for statistical analysis, and the significance of the difference between two groups was determined by Student’s *t*-test at *p* < 0.05, *p* < 0.01, and *p* < 0.001, respectively. Graphs were plotted using Origin 2018 software.

## 5. Conclusions

In this study, to enhance the levels of lipid and carotenoid in *Aurantiochytrium* sp., a total of four genes, including *crtW* and *crtZ* from *Haematococcus pluvialis*, *DGAT* from *H. Pluvialis* and *Phaeodactylum tricornutum*, and *LPAAT* from *P. tricornutum* and *Brassica napus*, were selected for heterologous expression in *Aurantiochytrium* sp. The expression of these genes directly or indirectly affected the pigment content in *Aurantiochytrium* sp. Transformant strain L-S of *Aurantiochytrium* sp. showed a significant increase in pigment content and altered fatty acid composition, with an increase in the relative abundance of unsaturated fatty acids. The results of transcriptomic analysis based on strain L-S revealed enhanced expression of fatty acid synthesis precursor acetyl coenzyme A and energy-supplying ATP-related metabolic processes, and the gene expression of *GGPS* and *PSY*, both encoding the key enzymes involved in the astaxanthin synthesis pathway, was up-regulated, ultimately affecting the carotenoid levels. In summary, this work demonstrated that metabolic engineering of *Aurantiochytrium* sp. could serve as a valuable and powerful tool for fundamental carotenoid research, enhancing our understanding of carbon metabolic flow from LPAAT to host cells and the synthesis of important metabolites, such as carotenoids and fatty acids.

## Figures and Tables

**Table 1:** Contents of astaxanthin and astaxanthin ester in wild type (WT) and transformant strains of *Aurantiochytrium* sp. Data represent the mean ± standard deviation (SD) of 3 biological replicates. Symbol “–” indicates no significant difference compared to the wild type (*p* > 0.05).

Strain	Astaxanthin Ester		Free Astaxanthin		Total Astaxanthin	
	Content (μg g^−1^)	Increase from WT (%) (*p* < 0.05)	Content (μg g^−1^)	Increase from WT (%) (*p* < 0.05)	Content (μg g^−1^)	Increase from WT (%) (*p* < 0.05)
WT	2.5 ± 0.0		5.9 ± 0.0		8.4 ± 0.0	
L-S	2.8 ± 0.9	–	8.9 ± 0.2	50.8%	11.7 ± 1.1	39.3%
L-Y	2.9 ± 0.5	–	6.4 ± 0.0	8.5%	9.3 ± 0.5	–
D-S	1.9 ± 0.0	–	5.7 ± 0.0	–	7.6 ± 0.0	–
2B	2.0 ± 0.2	–	6.0 ± 0.0	–	8.0 ± 0.1	–
CRTR-B1	4.7 ± 0.0	88.0%	6.9 ± 0.0	16.9%	11.5 ± 0.0	36.9%
CRTR-B2	3.3 ± 1.2	–	7.8 ± 0.0	32.2%	11.1 ± 1.2	32.1%
BKT3	4.1 ± 0.2	64.0%	5.8 ± 0.1	–	9.8 ± 0.3	–

**Table 2:** Contents of β-carotene and canthaxanthin in wild type (WT) and transformant strains of *Aurantiochytrium* sp. Data represent the mean ± standard deviation (SD) of 3 biological replicates. Symbol “–” indicates no significant difference compared to the wild type (*p* > 0.05).

Strain	β-Carotene		Canthaxanthin		Lutein	
	Content (μg g^−1^)	Increase from WT (%) (*p* < 0.05)	Content (μg g^−1^)	Increase from WT (%) (*p* < 0.05)	Content (μg g^−1^)	Increase from WT (%) (*p* < 0.05)
WT	88.0 ± 0.1		11.0 ± 0.1		20.3 ± 0.1	
L-S	461.7 ± 3.5	424.7%	110.2 ± 0.5	901.8%	137.2 ± 1.0	575.9%
L-Y	65.7 ± 0.8	–	19.4 ± 0.3	76.4%	31.8 ± 0.6	56.7%
D-S	57.0 ± 0.1	–	10.3 ± 0.1	–	12.0 ± 0.0	–
2B	91.6 ± 3.2	–	12.5 ± 0.4	13.6%	24.9 ± 0.5	22.7%
CRTR-B1	87.4 ± 0.8	–	13.1 ± 0.2	19.1%	31.8 ± 0.5	56.7%
CRTR-B2	84.5 ± 0.8	–	22.3 ± 0.3	102.7%	56.1 ± 0.7	176.4%
BKT3	112.0 ± 2.2	27.3%	18.6 ± 0.4	69.1%	19.3 ± 0.2	–

## Data Availability

All raw data are readily available upon request.

## Supplementary Materials

The following supporting information can be downloaded at: https://www.mdpi.com/article/10.3390/md23040164/s1, Figure S1: Quantitative real-time PCR analysis of genes *At3g50520*, *his7*, and *METK3* in wild type (WT) and strain L-S of *Aurantiochytrium* sp. Data are expressed as mean ± standard deviation (SD) of 3 biological replicates. The statistical differences are indicated by *p* < 0.001 (***); Figure S2: Carotenoid content of wild type (WT) and transformant strains of *Aurantiochytrium* sp. under the induction of methanol or ethanol. (a) Astaxanthin content under methanol induction. (b) Astaxanthin content under ethanol induction. (c) β-carotene content under methanol induction. (d) β-carotene content under ethanol induction. (e) Canthaxanthin content under methanol induction. (f) Canthaxanthin content under ethanol induction. Data are expressed as mean ± standard deviation (SD) of 3 biological replicates. Different lowercase letters “a, b, c, d, e, and f” or “A, B, C, D, E, and F” represent significant differences between treatments (*p* < 0.05); Table S1: Primer and their sequences used in the present study; Table S2: Primers used for genes validated by quantitative real-time PCR.
