# Supplementary material for: Effect of Heterologous Expression of Key Enzymes Involved in Astaxanthin and Lipid Synthesis on Lipid and Carotenoid Production in Aurantiochytrium sp
Source: Mar Drugs. 2025 Apr 11;23(4):164. doi: 10.3390/md23040164 (PMC12028430; doi:10.3390/md23040164)
Supplement: Supplementary file 1 [file marinedrugs-23-00164-s001.zip › marinedrugs-3464937-supplementary.pdf]

Table S1. Primer and their sequences used in the present study.

Primer	Sequence (5'→3')
PMG-F	TTGTTTAAACGAGCTCGCTAGCCT
PMG-R	GGTTTAGTTCCTCACCTTGTCGTATTATACT
D-S-F	ATACGACAAGGTGAGGAACTAAACCATGGCCCAAGCCATGCCT
D-S-R	gaggctagcgagctcgtttaaacaaTCAATGGTGGTGATGATGGTGAC
2B-F	ATACGACAAGGTGAGGAACTAAACCATGGGTGTCGCAACGAATGC
2B-R	gaggctagcgagctcgtttaaacaaTCAATGGTGGTGATGATGGTGC
L-Y-F	ATACGACAAGGTGAGGAACTAAACCATGGCTATGGCCGCTGCTG
L-Y-R	gaggctagcgagctcgtttaaacaaTCAGTGGTGGTGGTGGTGGTGCT
L-S-F	ATACGACAAGGTGAGGAACTAAACCATGTTCGCCGCCATGAACG
L-S-R	gaggctagcgagctcgtttaaacaaTTAATGGTGGTGATGATGGTGAGATG
BKT3-F	ATACGACAAGGTGAGGAACTAAACCATGcatcaccatcatcaccatCAAG
BKT3-R	gaggctagcgagctcgtttaaacaaTCATGCCAAGGCAGGCACCAGGCCAC
CRTR-B1-F	ATACGACAAGGTGAGGAACTAAACCATGCATCATCACCATCACCACC TG
CRTR-B1-R	gaggctagcgagctcgtttaaacaaCTACCGCTTGGACCAGTCCAGTTC
CRTR-B2-F	ATACGACAAGGTGAGGAACTAAACCATGcatcaccatcatcaccatGCTTTG
CRTR-B2-R	gaggctagcgagctcgtttaaacaaTCACACCCTGCGCTTTGACCAGTC

Table S2. Primers used for genes validated by quantitative real-time PCR.

Primer	Sequence (5'→3')
18S-F	CGCAGCAGCACATGAGAAA
18S-R	TTCAGCCTTGCGACCATACTC
At3g50520-F	CCTGTTTACCACCGACACGA
At3g50520-R	GACCCTGAAAAACCCGCAAC
his7-F	CCGGCATTTTGCCATCAACA
his7-R	CGAGATTCGAGCGTACGGAA
METK3-F	CAAGAACGTCGGCTACACCT
METK3-R	CAATATCGGGGGACTGCTCC

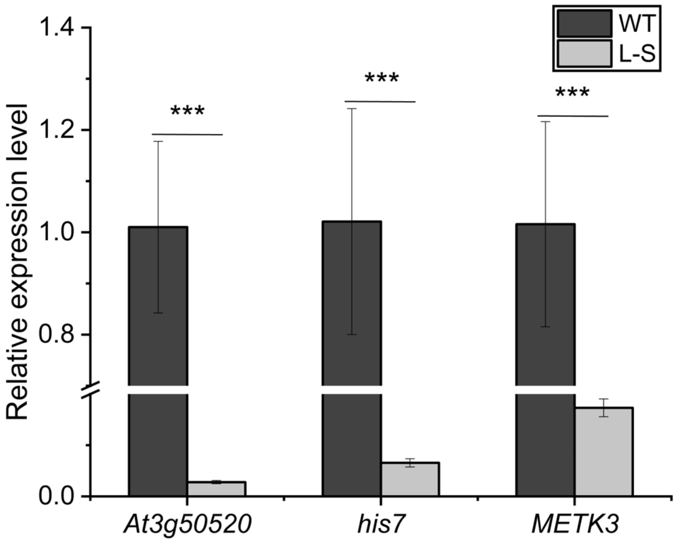

Figure S1. Quantitative real-time PCR analysis of genes *At3g50520*, *his7*, and *METK3* in wild type (WT) and strain L-S of *Aurantiochytrium* sp. Data are expressed as mean ± standard deviation (SD) of 3 biological replicates. The statistical differences are indicated by $P < 0.001$ (***).

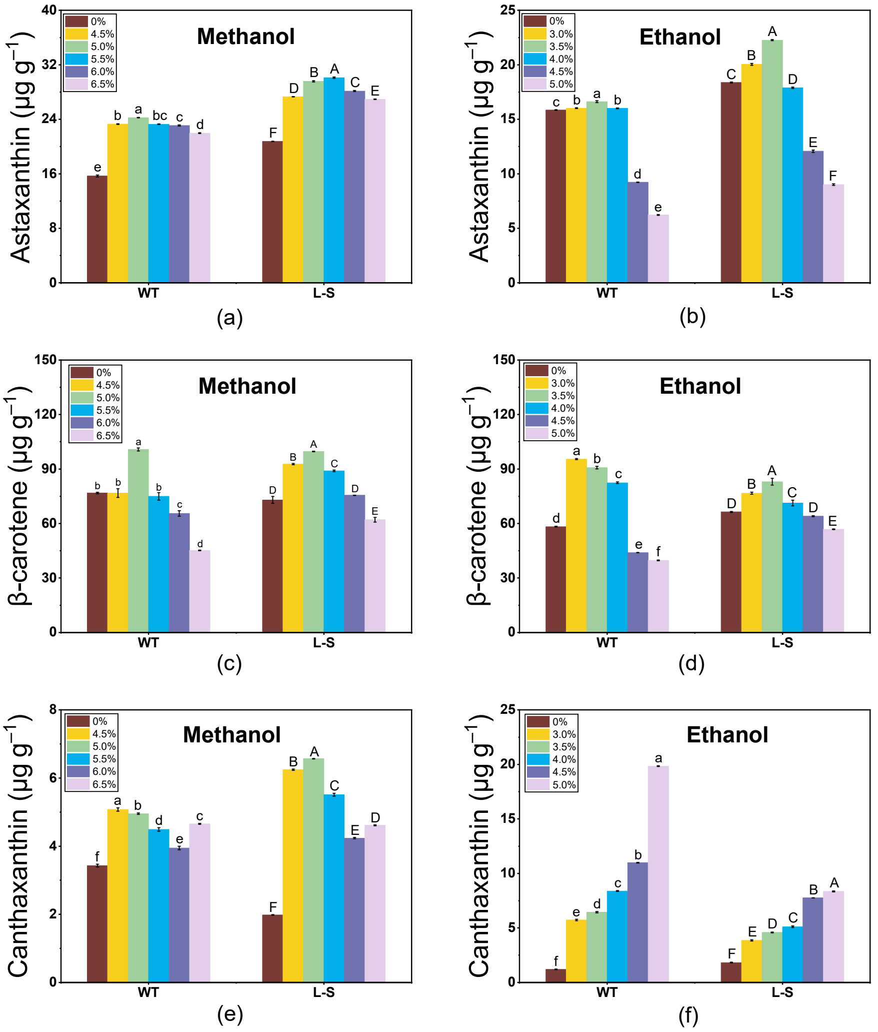

Figure S2. Carotenoid content of wild type (WT) and transformant strains of *Aurantiochytrium* sp. under the induction of methanol or ethanol. (**a**) Astaxanthin content under methanol induction. (**b**) Astaxanthin content under ethanol induction. (**c**) β-carotene content under methanol induction. (**d**) β-carotene content under ethanol induction. (**e**) Canthaxanthin content under methanol induction. (**f**) Canthaxanthin content under ethanol induction. Data are expressed as mean ± standard deviation (SD) of 3 biological replicates. Different lowercase letters "a, b, c, d, e, and f" or "A, B, C, D, E, and F" represent significant differences between treatments ($P < 0.05$).
